# A Novel Organic Electrochemical Transistor-Based Platform for Monitoring the Senescent Green Vegetative Phase of *Haematococcus pluvialis* Cells

**DOI:** 10.3390/s17091997

**Published:** 2017-08-31

**Authors:** Weiwei Wei, Kang Xiao, Ming Tao, Lifu Nie, Dan Liu, Shanming Ke, Xierong Zeng, Zhangli Hu, Peng Lin, Yu Zhang

**Affiliations:** 1 Shenzhen Key Laboratory of Special Functional Materials & Guangdong Research Center for Interfacial Engineering of Functional Materials, College of Materials Science and Engineering, Shenzhen University, Shenzhen 518060, China; weiwei2011@semi.ac.cn (W.W.); 2150120413@email.szu.edu.cn (L.N.); 2150120415@email.szu.edu.cn (D.L.); smke@szu.edu.cn (S.K.); zengxier@szu.edu.cn (X.Z.); 2 Key Laboratory of Optoelectronic Devices and Systems of Ministry of Education and Guangdong Province, College of Optoelectronic Engineering, Shenzhen University, Shenzhen 518060, China; boxiaokang@szu.edu.cn (K.X.); 2156180101@email.szu.edu.cn (M.T.); 3 Shenzhen Key Laboratory of Marine Bioresource and Eco-environmental Science, Guangdong Engineering Research Center for Marine Algal Biotechnology, College of Life Sciences and Oceanography, Shenzhen University, Shenzhen 518060, China; huzl@szu.edu.cn

**Keywords:** organic electrochemical transistor, real-time, senescent green vegetative phase, *Haematococcus pluvialis*

## Abstract

The freshwater unicellular microalga *Haematococcus pluvialis* (*H. pluvialis*) has gained increasing attention because of its high-value metabolite astaxanthin, a super anti-oxidant. For the maximum astaxanthin production, a key problem is how to determine the senescent green vegetative phase of *H. pluvialis* cells to apply the astaxanthin production inducers. The conventional methods are time-consuming and laborious. In this study, a novel platform based on organic electrochemical transistor (OECT) was produced. A significant channel current change of OECTs caused by settled *H. pluvialis* cells on the poly(3,4-ethylenedioxythiophene): polystyrene sulfonate (PEDOT: PSS) film was recorded commencing from 75 min and a stationary stage was achieved at 120 min after the combined treatment of blue light irradiation and sodium bicarbonate solution additives, which indicate the onset and maturation of the senescent green vegetative phase, respectively. Therefore, the appropriate time point (120 min after sample loading) to apply astaxanthin production inducers was determined by as-fabricated OECTs. This work may assist to develop a real-time biosensor to indicate the appropriate time to apply inducers for a maximum astaxanthin production of *H. pluvialis* cells.

## 1. Introduction

Sensitively and rapidly detecting analytes in solution is a pressing need in areas of biology, chemistry and medicine. Traditional laboratory techniques, such as microscopic observation or secondary fluorescent labels, have great sensitivity and accuracy. However, these experimental processes have significantly high cost and complexity and are incapable of monitoring the real-time interactions between probe and analyte target. Thus, extensive research has been conducted to develop novel sensor platforms which can conveniently and efficiently detect the targets in a real-time manner. Among these devices, organic electrochemical transistor (OECT), a new branch of organic thin-film transistor (OTFT), is gaining increasing attention in biological and chemical detections due to its advantages in fabrication, performance, biocompatibility, expense, and so on. Employing conducting polymer in an electrolyte-gated structure [1], the working mode of OECT is based on the regulation of active layer’s conductivity by the chemical doping of cations from the electrolyte to the channel film under a gate voltage [2]. Therefore, ions [3,4], proteins [5], DNA [6], dopamine [7], lactate [8], and glucose [9,10,11] have been successfully detected by OECT-based sensors. In particular, OECTs have shown promising results in active control of cell growth [12], monitoring the cell morphology changes [13], and recording the tissue activity [14] with high sensitivity and speediness.

Microalgae cells have been broadly studied for a variety of applications, such as the production of high-value metabolites [15] and wastewater treatment [16,17]. *Haematococcus pluvialis* (Chlorophyceae) is a freshwater unicellular microalga, which produces astaxanthin with 20 times greater antioxidant capacity than that produced by chemical synthesis [18]. Normally, *H. pluvialis* is studied for its adaptive response under conditions of environmental stress such as nutrient depletion, high salinity, or high light intensities. In favorable or adverse environment, there are two morphologically distinguishable phases in the life cycle of *H. pluvialis*, green vegetative phase and red non-motile astaxanthin accumulated phase (red cyst phase or aplanospore) [19]. As a large amount of astaxanthin accumulates in cellular lipid droplets in the aplanospore stage, plenty of efforts have been put into maximizing the biomass and astaxanthin production in *H. pluvialis* for commercial purposes. Many studies focused on optimizing the culture parameters, including nutrient compositions, chemical treatment, light, and so on [19].

A two-stage cultivation of *H. pluvialis* for producing astaxanthin has commonly been utilized, i.e., growth and production stages. It has been reported that the optimal induction stage for the highest astaxanthin production of *H. pluvialis* was in senescent green vegetative phase of the growth stage, in which green cells lose the mobility and turn into settled cysts [20]. A critical question is how to determine the appropriate time point to apply the astaxanthin production inducers to transfer cells into the second production stage for maximum astaxanthin production. This determination is required for every batch of astaxanthin production because the different algal statuses among batches of *H. pluvialis* cells often cause the variation of the appropriate induction time point. However, the current method of determining senescent green vegetative phase, which is based on microscopic observation or optical density measurement, is a time-consuming and laborious process. It is of great necessity to develop novel platforms that allow sensitive and real-time detection of the stage transition of *H. pluvialis*.

This study presents the first report to apply OECTs in the detection of microalgae *H. pluvialis* cells. A convenient, efficient, and high-throughput measuring platform based on OECT array was constructed. *H. pluvialis* cells were real-time monitored from early green vegetative phase and the timeline of the senescent green vegetative phase was determined by the channel current change of OECTs caused by settled *H. pluvialis* cells on the transistors. These results may assist in developing a real-time sensor to indicate the appropriate time point to apply astaxanthin production triggers for a maximum astaxanthin production in a commercial fermenter.

## 2. Materials and Methods

### 2.1. Materials

PEDOT:PSS (Poly(3,4-ethylenedioxythiophene) polystyrene sulfonate) was purchased from Sigma-Aldrich (St Louis, MO, USA). PDMS-Sylgard Elastomer 184 was obtained from Dow Corning (Midland, TX, USA). The recommended ESP Ag culture medium for *H. pluvialis* cultivation was used, which is composed of KNO_3_ (0.2 g·L^−1^), K_2_HPO_4_ (0.02 g·L^−1^), MgSO_4_ (0.02 g·L^−1^), soil extract (3%, *v/v*), micronutrient solution (0.5%, *v/v*) and proteose-peptone (0.1%, *w/v*). Sodium bicarbonate and other unspecified general chemicals were purchased from Aladdin (Shanghai, China).

### 2.2. Device Fabrication

The fabrication process was performed according to the previous report with a slight modification [21]. Briefly, SU-8 was used for patterning the PEDOT:PSS layer, insulating the underlined metal electrodes from electrolytes and trapping the algae cells in confined space, instead of PEG (poly(ethylene glycol)) in the report of Zhang et al. [21] for modification. Firstly, OECT array was fabricated on a glass substrate with a size about 3 cm × 3 cm and a thickness about 0.1 mm. Cr/Au film with 10/100 nm was deposited on the glass substrate through a thermal evaporation (NEXDEP, The AE Inc., Kitchener, ON, Canada) and patterned for source and drain electrodes by lift-off process. The width and length of the channel were 500 and 20 μm, respectively. Then SU-8 microwells were fabricated by photolithography, and a designed size of about 500 μm × 500 μm was achieved. PEDOT:PSS was spin-coated on the surface of SU-8, and the thickness was about 80 nm which was characterized by a scanning probe microscope. Then, PEDOT:PSS was physically delaminated from the surface of SU-8 by scotch tape, leaving only 500 μm × 500 μm patterns in SU-8 microwells. The specimen was then annealed at 180 °C for 1 h in a high-purity N_2_ filled glove box. Finally, Polydimethylsiloxane (PDMS) walls were fabricated by soft-lithography [22]. PDMS polymer in a ratio of 10:1 was poured into a stainless steel mould with micro pillar array, treated at 85 °C for 1 h in an oven, and then peeled off from the mould. The diameter and depth of the formed PDMS walls were 4 mm and 1 cm, respectively. At last, PDMS walls were bonded with glass substrate after oxygen plasma treatment for 30 s.

### 2.3. Device Characterization and Image Acquisition

OECT array characteristics (transfer characteristics, output characteristics and the real-time dynamic response) were measured by using a semiconductor parameter analyzer Keithley 4200S. A commercial Pt wire (φ 0.5 mm × 37 mm, Gaoss Union, Wuhan, China) immersed in the electrolyte was used as the gate electrode. The transfer characteristics were obtained by measuring the change of channel current *I*_DS_ as a function of gate voltage *V*_G_ when drain voltage *V*_DS_ was kept at 0.1 V. The output characteristics were obtained by measuring the change of the channel current *I*_DS_ as a function of drain voltage *V*_DS_ under a stepped gate voltage *V*_G_ of 0, 0.2, 0.4, 0.6 and 0.8 V, respectively, leading to different curves of *I*_DS_ versus *V*_DS_. To real-time monitor the settlement of *H. pluvialis* cells in the senescent green vegetative phase, a dynamic response of *I*_DS_ versus time *t* was measured.

The images of settled *H. pluvialis* cells were taken by a stereomicroscope equipped with a cooled CCD (Charge-coupled Device) camera (Olympus SZX16, Tokyo, Japan) after on-chip assay. The acquired image data were further processed by Adobe Photoshop (Adobe Systems).

### 2.4. H. pluvialis Preparation

*H. pluvialis* cells were cultured according to the method described by Zheng [23]. Briefly, Green alga *H. pluvialis* 192.80 was purchased from the SAG (Sammlung von Algenkulturen Göttingen), Germany. The algal cells were grown in 100 mL ESP Ag culture medium exposed to light with the intensity of 22.5 μmol·photon·m^−2^·s^−1^ at 21 °C.

Once *H. pluvialis* cells reached 7 d green phase, the exposure to blue light with the intensity of 22.5 μmol·photon·m^−2^·s^−1^ generated by a home-made fluorescent lamp box and the addition of NaHCO_3_ (0.5 mM) were both utilized to trigger cells to enter the senescent green vegetative phase. Under this combined treatment, 100 μL *H. pluvialis* cells were loaded into each as-fabricated OECT to monitor the timeline of the senescent green vegetative phase.

## 3. Results and Discussion

### 3.1. Preparation and Characterization of OECT Array

**Figure 1:**
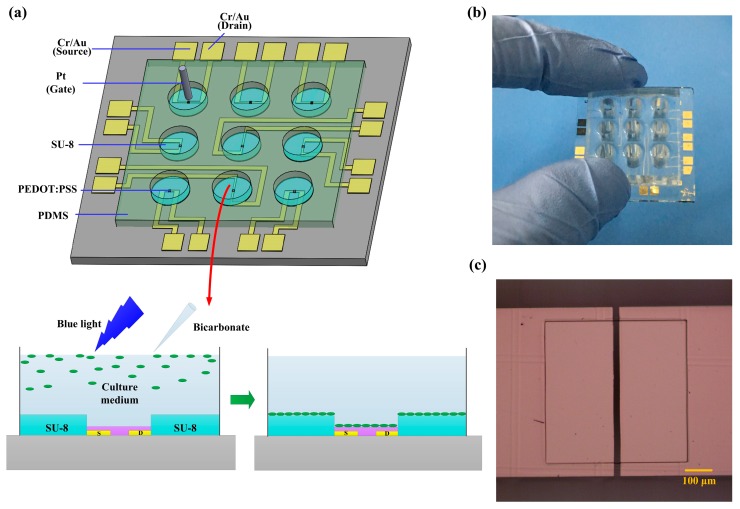
(**a**) The schematic diagram of an OECT array-based measuring platform. For the platform, the yellow region represented Cr/Au electrodes, the grey pillar represented gate electrode, the blue region represented SU-8, the pink region represented PEDOT:PSS, the green region represented PDMS, and the grey block represented glass substrate, the OECT device was characterized before and after the transition of *H. pluvialis* from motile to precipitate phase, in which the culture medium served as the electrolyte; (**b**) Photograph of the OECT array platform; (**c**) Microscopic image of the SU-8 microwell.

Figure 1a described the schematic diagram of OECT array-based measuring platform (a photograph of 3 × 3 OECT array platform fabricated on a glass substrate shown in Figure 1b). PEDOT:PSS film, a kind of conducting polymer with excellent biocompatibility [24], was used as the active layer of OECT device array. The patterned film of Cr/Au served as the source and drain electrodes, insulated from the electrolyte by SU-8 layer. A microscopic image of the SU-8 well was shown in Figure 1c. The culture medium in the PDMS walls was used as the electrolyte, and a commercial Pt wire immersed in the electrolyte was used as the gate electrode. In the lower panel of Figure 1a, it showed the measurement schema. Before treatment, *H. pluvialis* cells in green vegetative phase were motile in the favorable cultivation. When combined treatment of blue light irradiation and sodium bicarbonate solution additives were applied for 2 h, the green motile algal cells would turn to be non-motile and prone to precipitate in the stressful cultivation. After the precipitated algal cells with electric charges attached on the OECT device, the channel current of OECT would change, suggesting the onset of the senescent green vegetative phase.

**Figure 2:**
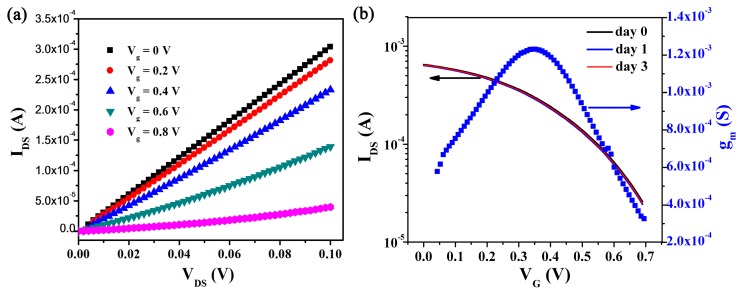
Characteristics of the OECT device array. (**a**) Output characteristic of a single OECT measured in culture medium; (**b**) Transfer characteristic of a single OECT measured in culture medium in 3 days at *V*_DS_ = 0.1 V and the resulting transconductance *g*_m_.

The OECT device array in the measuring platform was characterized in culture medium with Pt wire as the gate electrode. Without a gate dielectric layer, the conducting active layer direct interfaced with the electrolyte solution, OECT thus could work in a low-voltage operation (below 1 V), as depicted by output characteristic of the device in Figure 2a. The low working voltage feature is crucial for cell sensing, as it can avoid the detrimental effects induced by water hydrolysis to the cells. The stability of the device in culture medium was also critical for functioning as a cell-based biosensor. The device showed a constant stability during the measuring period (3 days) in culture medium (Figure 2b), which ensured a reliable recording. OECTs operated in a depletion mode since the density of holes in the PEDOT:PSS film was influenced by chemical doping [2]. The channel current *I*_DS_ had a nearly 2 orders magnitude of modulation at only 0.7 V of gate voltage *V*_G_. A maximal transconductance *g*_m_ of 1.2 ms was obtained at 0.32 V of *V*_G_, which means a high gain for transistors in sensing applications, as these OECTs had a high ratio of response output to signal input.

### 3.2. Real-Time Algal Cells Detection

The application of as-fabricated OECT arrays in the detection of the senescent green vegetative phase was performed into two steps: First, a one-shot test to examine the impact of cell settlement on the device performance by detecting settled *H. pluvialis* cells in the senescent green vegetative phase (or green cyst phase); Second, a real-time detection of settled *H. pluvialis* cells in the senescent green vegetative phase after the combined treatment of blue-light and sodium bicarbonate.

**Figure 3:**
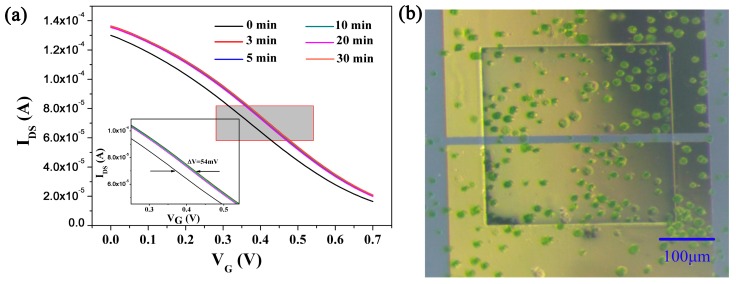
(**a**) *I*_DS_ vs *V*_G_ (transfer characteristics) of an OECT measured in the culture medium before (0 min) and at 3, 5, 10, 20 and 30 min after loading the green cyst *H. pluvialis* cells, *V*_DS_ = 0.1 V; (**b**) Optical image of the green cyst *H. pluvialis* cells precipitated on the OECT channel film in the SU-8 microwell.

In the first test, the correlation between channel current change of as-fabricated OECTs and the precipitation of *H. pluvialis* cells in green cyst phase was demonstrated, verifying the feasible application of as-fabricated OECTs in detection of cell settlement on the device. Cells in green cyst phase were loaded into the platform, precipitate cells would rapidly settle on PEDOT:PSS film of OECTs. We found that the transfer characteristic shifted to larger gate voltage in a three-minute delay, and then became stable, suggesting that the green cyst cells settled completely in a minute scale. This shift caused by the settled green cyst cells was measured as 54 mV (Figure 3a). As shown in Figure 3b, the settlement of green cyst cells on the OECT channel film was confirmed by stereomicroscope after on-chip test. As the control group, the cell-free culture medium could not change the performance of the OECT device, indicating that the change was indeed induced by cell settlement on the PEDOT:PSS film. Also, this response was detected in a minute scale, suggesting a rapidity of as-fabricated OECT arrays to the settlement of *H. pluvialis* cells.

To investigate the influence of algal cells settlement on the device performance, it is necessary to investigate the electrical interaction of the algal cell with our OECT device. The channel current *I*_DS_ of OECT is as follows:

(1) $$\begin{array}{l} I_{\mathrm{DS}}=\frac{q\mu p_{0}tW}{LV_{p}}(V_{p}-V_{G}^{\mathrm{eff}}+\frac{V_{\mathrm{DS}}}{2})V_{\mathrm{DS}}\;(\mathrm{when}\;\left|V_{\mathrm{DS}}\right|<<\left|V_{p}-V_{G}^{\mathrm{eff}}\right|)\\ V_{p}=qp_{0}t/c_{i}\\ V_{G}^{\mathrm{eff}}=V_{G}+V_{\mathrm{offset}} \end{array}$$

where *q* is the charge of electron, *μ* is the mobility of vacancy, *P*_0_ is the initial hole concentration, *t* is the channel film thickness, *L* and *W* are the channel length and width, respectively. *V*_p_ represents the pinch-off voltage, and *c*_i_ is the unit area capacitance of the transistor, which is related to the channel-electrolyte capacitance (*C*_c_) and the gate-electrolyte capacitance (*C*_G_) [25]. $V_{G}^{\mathrm{eff}}$ is an effective applied gate voltage, and *V*_offset_ is the offset voltage, which represents the potential drop at the channel-electrolyte and gate-electrolyte interfaces [11,26]. According to our previous experience [13], *c*_i_ changed very slightly after cells were cultivated on the PEDOT:PSS film of OECT. Therefore, the change of the offset voltage *V*_offset_ was the main reason for the shift of transfer curve.

**Figure 4:**
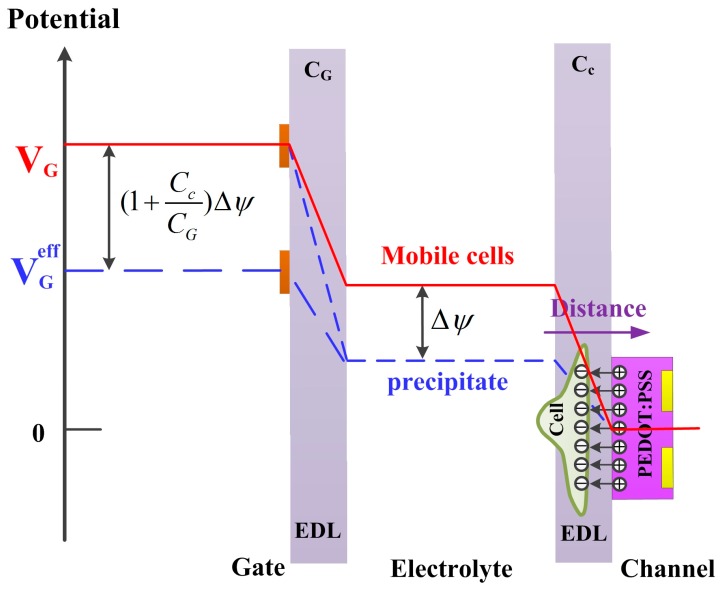
Schematic diagram of potential distribution between the gate electrode interface and the channel interface in the presence and absence of the *H. pluvialis* cells attachment. EDL is representative of electric double layer, including the electric double layer of channel-electrolyte and gate-electrolyte.

The analytical potential model before and after cell settlement on an OECT device can be shown in Figure 4. In the first beginning, there was no cell coverage on the device. The applied gate voltage distributed on the two interfaces of channel-electrolyte and gate-electrolyte (the solid line in Figure 4), and the voltage *V*_E-C_ distributed at the channel-electrolyte interface is given as:

(2) $$V_{C-E}=\frac{V_{G}}{1+\gamma }$$

where $\gamma =C_{c}/C_{G}$. Then, the green cyst *H. pluvialis* cells precipitated quickly onto the PEDOT:PSS film, so there would be a potential change Δψ at the electrolyte/PEDOT:PSS interface [13]. Therefore, the offset voltage change is expressed as:

(3) $$\Delta V_{\mathrm{offset}}=(1\;+\gamma )\Delta \psi$$

Since there is nearly no change for *c*_i_, γ can approximately be considered as a constant value in this experiment [13,27]. Thus, $\Delta V_{\mathrm{offset}}$ is only related to the potential change Δψ. Δψ is normally modulated by the electrostatic interaction between the algal cells and the OECT device [28]. It is reasonable to assume that there is an electric potential drop related to the distance between the cells and PEDOT:PSS film, and the potential drop is called Zeta potential. In cell biology, the Zeta potential is normally related to electrostatic adhesion [28], cell agglutination and biological activation [29]. Then the potential change Δψ can be expressed as an average Zeta potential of the settled algal cells, which is given as the following [30,31]:

(4) $$\zeta =\frac{Q}{\varepsilon Dr^{2}}\times \frac{1}{\kappa }$$

where *Q* is the surface charge of the algal cell, ε is the electrolyte permittivity, *D* is the dielectric displacement, *r* is radius of the algal cell, 1/κ is the double layer length. Thus, the cell Zeta potential is modulated by the cells surface charge density and the ion types and concentration in the electrolyte.

In our OECT array, γ is about 0.34 as reported previously [27]. Since the offset voltage change was −54 mV, the potential change Δψ could be calculated as −40.3 mV from Equation (3). For the zeta potential determination, it was measured by using a Zeta potential analyzer (ZETASIZER NANO, Malvern, UK) (see Figure S1 in the Supplementary Materials). From the measurement, the Zeta potential of the *H. pluvialis* cells was in the range of −20.7 mV to −32.3 mV. In addition, many other researchers reported the Zeta potential of algal cells with a value from −15 to −40 mV [32,33,34,35]. The measurement and reports were in the similar range of the OECT potential change Δψ −40.3 mV.

As mentioned above, the Zeta potential of the *H. pluvialis* cells is in negative tens of millivolts [28,30]. When algal cells settled on the PEDOT:PSS film, the potential drop *V*_C-E_ at the channel-electrolyte interface would decrease under a fixed gate voltage *V*_G_. Since the *V*_C-E_ directly induced the modulation of the channel current of the OECT device, it could be considered that the effective gate voltage decreased, as shown in Figure 4 (dashed line). Therefore, it required a larger gate voltage to compensate the reduced effective gate voltage. As a result, the transfer characteristic shifted to a higher value, which was consistent with the results shown in Figure 3a. Furthermore, the algal cells indeed applied an additional voltage of negative tens of millivolts, confirming the above explanation.

**Figure 5:**
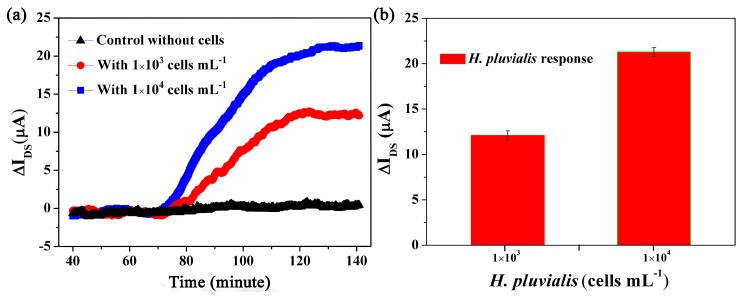
Timeline of the senescent green vegetative phase recorded by OECT. (**a**) In situ OECT response with (red and blue) and without (black) *H. pluvialis* cells under the light and salt treatment. The real-time transistor current was converted to current change for uniformity, *V*_G_ = 0.3 V, *V*_DS_ = 0.1 V. (**b**) Recorded current changes in response to different concentrations of *H. pluvialis*. Each concentration was repeated five times.

As mentioned, for maximum astaxanthin production, the production inducers should be applied in the senescent green vegetative phase of the growth stage of *H. pluvialis* cells. It is of high significance to determine the timeline of the senescent green vegetative phase. Therefore, in a second test, we performed the real-time detection of the senescent green vegetative phase by using as-fabricated OECTs to determine the appropriate time point to apply astaxanthin production inducers. According to our unpublished results, green motile *H. pluvialis* cells at Day 7 cultivation were observed by microscopic examination to enter the senescent green vegetative phase within around 1 h after the combined treatment of blue light (22.5 μmol·photon·m^−2^·s^−1^) and NaHCO_3_ (0.5 mM). Under this combined treatment, *H. pluvialis* cells were able to be completely converted into precipitate green cyst cells within 2 h. In the OECT device tests, cell-free culture medium and two different densities of *H. pluvialis* cells (1 × 10^3^ and 1 × 10^4^ cells·mL^−1^) were assayed as control and experimental groups, respectively. All groups were treated in the home-made fluorescent lamp box with blue light (22.5 μmol photon m^−2^·s^−1^) and NaHCO_3_ (0.5 mM) upon sample loading. Figure 5a depicts a typical dynamic response of OECT after the treatment. In order to eliminate the variations between different devices, the real-time current records were converted into current change Δ*I*_DS_, and the current obtained in control groups was fitted to a baseline for uniformity. As shown in Figure 5a, in comparison with the largely unchanged baseline given by cell-free culture medium, the dramatic risings of channel current were observed at about 75 min after sample loading of *H. pluvialis* cells. This current rising lasted for around 45 min followed by a stationary stage. Notably, the current changes were different, i.e., around 12.1 μA and 21.4 μA (Figure 5b), respectively, as cell densities varied. An explanation for these observations was given as follows. Within 75 min after sample loading, *H. pluvialis* cells in green vegetative phase gradually lost motility and a small proportion of *H. pluvialis* cells started to precipitate and attach on the OECT device, thus the channel current of OECT remained largely unchanged or increased very slowly. After about 75 min, more and more cells began to precipitate and attached on the OECT device to cause a significant channel current rising, indicating the onset of the senescent green vegetative phase. The stationary stage starting at around 120 min represented the completion of cell settlement. These results were consistent with the above-mentioned unpublished observation that green motile *H. pluvialis* cells entered the senescent green vegetative phase within around 1 h and then completely precipitated within 2 h after the combined treatment of blue light (22.5 μmol photon m^−2^·s^−1^) and NaHCO_3_ (0.5 mM). Therefore, under our tested conditions, the appropriate time point (120 min after sample loading) to apply astaxanthin production inducers was determined by as-fabricated OECTs. This may assist in indicating the appropriate time to apply inducers for a maximum astaxanthin production of *H. pluvialis* cells.

## 4. Conclusions

In summary, we applied PEDOT:PSS-based OECT array to detect microalgae *H. pluvialis* cells for the first time. The settlement of *H. pluvialis* cells in senescent green vegetative phase was successfully monitored by the OECT-based platform for a real-time electrical detection. Under the combined treatment of blue light and NaHCO_3_ salt, a significant channel current change of OECTs caused by settled *H. pluvialis* cells on the PEDOT:PSS film was recorded commencing from 75 min and a stationary stage was achieved at 120 min after sample loading, indicating that the onset and maturation of the senescent green vegetative phase occurred at 75 and 120 min after sample loading, respectively. Therefore, the appropriate time point (120 min after sample loading) to apply astaxanthin production inducers has been determined. With the ease of the real-time electrical readout and simple configuration of our device, it has the potential to develop a real-time biosensor to indicate the appropriate time to apply for a maximum astaxanthin production of *H. pluvialis* cells. In future, a high-throughput assessment on the appropriate application time of different inducers for astaxanthin production by *H. pluvialis* cells can be easily achieved by OECT arrays. Furthermore, our OECTs can be applied into broad applications of biological studies. For instance, the settlement of the marine biofouling organisms can be real-time monitored and quantified to develop an automatic anti-fouling drug screening platform based on as-fabricated OECT arrays.

## Supplementary Materials

The following are available online at http://www.mdpi.com/1424-8220/17/9/1997/s1, Figure S1: The Zeta potential recorded every 10 min for 2 h, Figure S2. Raw current trace data with (red and blue) and without (black) H. pluvialis cells under the light and salt treatment recorded by OECT. VG = 0.3 V, VDS = 0.1 V, Figure S3. (a) Device characteristics in the culture medium without algae before and after addition of sodium bicarbonate and blue light irradiation.; (b) Device characteristics in the culture medium with the attached green cyst cells before and after addition of sodium bicarbonate and blue light irradiation, Figure S4. Device characteristics in the culture medium with the salt and light irradiation with and without algae cells at typical time points of (a) 0 min, (b) 90 min and (c) 120 min, Figure S5. Transfer characteristics of the OECT in the culture medium with sodium bicarbonate initially and at 3 min and 2 h after loading the green cyst *H. pluvialis* cells, *V*_DS_ = 0.1 V, Figure S6. In situ OECT response with H. pluvialis cells for 2 h without the light and salt treatment, VG = 0.3 V, VDS = 0.1 V.
