# Supplementary material for: A Novel Organic Electrochemical Transistor-Based Platform for Monitoring the Senescent Green Vegetative Phase of Haematococcus pluvialis Cells
Source: Sensors (Basel). 2017 Aug 31;17(9):1997. doi: 10.3390/s17091997 (PMC5620665; doi:10.3390/s17091997)
Supplement: Supplementary file 1 [file sensors-17-01997-s001.pdf]

Supplementary Materials: A Novel Organic Electrochemical Transistor Based Platform for Monitoring the Senescent Green Vegetative Phase of *Haematococcus Pluvialis* Cells

Weiwei Wei, Kang Xiao, Ming Tao, Lifu Nie, Dan Liu, Shanming Ke, Xierong Zeng, Zhangli Hu, Peng Lin, Yu Zhang

For the Zeta potential measurement, a Zeta potential analyzer (ZETASIZER NANO, Malvern) was used. The green cyst *H. pluvialis* cells were loaded in a cuvette (particle number n=1000), and the Zeta potential was recorded every 10 min for 2 h. As shown in Figure S1, the Zeta potential of the *H. pluvialis* cells was in the range of -20.7 mV to -32.3 mV.

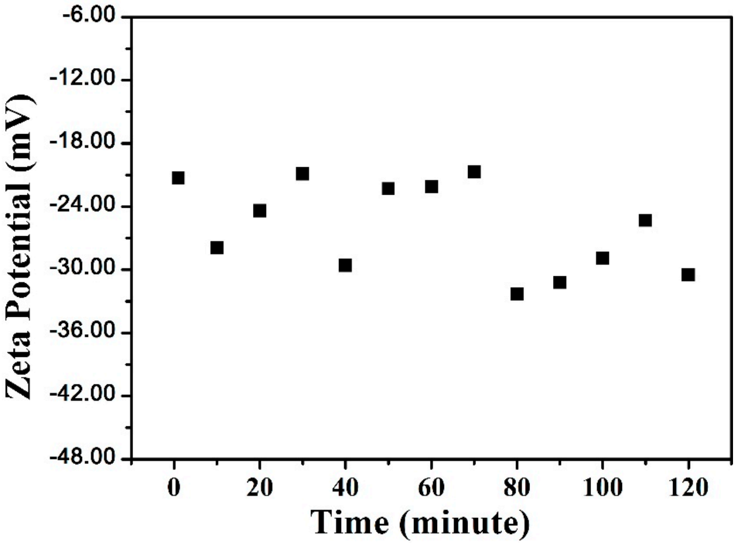

Figure S1. The Zeta potential recorded every 10 min for 2 h.

Figure S2 shows the real time OECT response of current trace with and without the *H. pluvialis* cells attachment on the channel film, which were smoothed and converted into current change ΔI_{DS} in Figure 5a, and the current obtained in control groups was fitted to a baseline for uniformity.

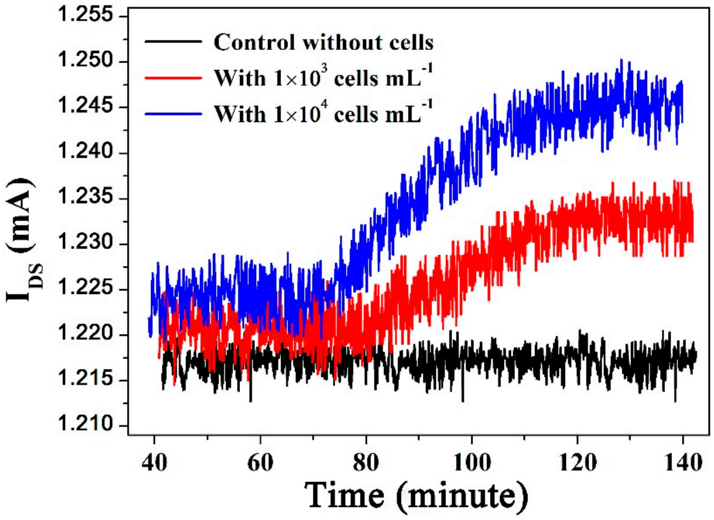

Figure S2. Raw current trace data with (red and blue) and without (black) *H. pluvialis* cells under the light and salt treatment recorded by OECT. $V_G = 0.3$ V, $V_{DS} = 0.1$ V.

In order to verify the influence of the sodium bicarbonate and blue light irradiation on the device performance, transfer characteristics of the OECT were measured in the culture medium without algae before and after addition of sodium bicarbonate and blue light irradiation. The results are shown in figure S3.

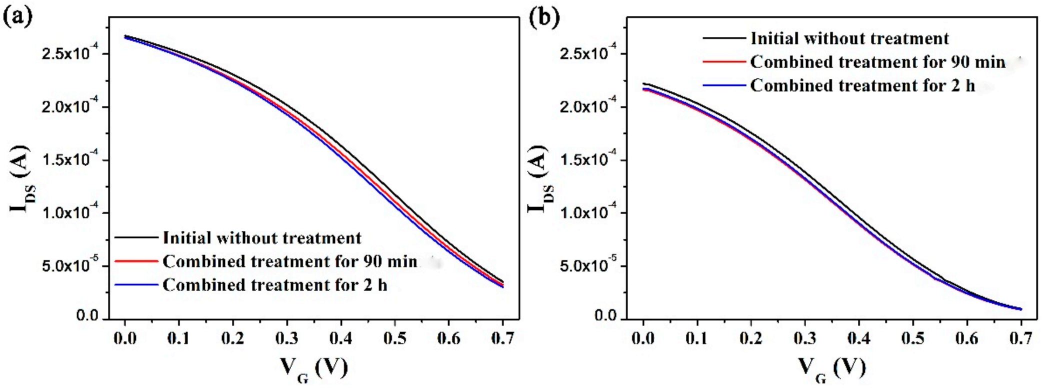

Figure S3. (a) Device characteristics in the culture medium without algae before and after addition of sodium bicarbonate and blue light irradiation.; (b) Device characteristics in the culture medium with the attached green cyst cells before and after addition of sodium bicarbonate and blue light irradiation.

As shown in Figure S3a, there was a tiny shift of the transfer curve to low gate voltage horizontally. This was mainly because the cations in sodium bicarbonate solution, such as Na^+ and H^+, could be injected into the PEDOT:PSS layer under a positive gate voltage and decrease the hole density as well as the channel conductance of the OECT. It would shift more with the increase of the concentration of cations. However, because the culture medium also contained some cations, such as K^+, H^+, and Mg^{2+}, the salt of sodium bicarbonate had little influence on the device response. As shown in Figure S3b, there was also a tiny shift of the transfer curve to low gate voltage horizontally. In all, the sodium bicarbonate and blue light irradiation had little influence on the device response.

Meanwhile, the transfer characteristics of the OECT in the solution containing sodium bicarbonate and blue light irradiation with and without the green motile algae cells at some typical time point were also supplemented (the concentration of 1×10^3 cells/mL was selected as an example), and the results were shown in Figure S4.

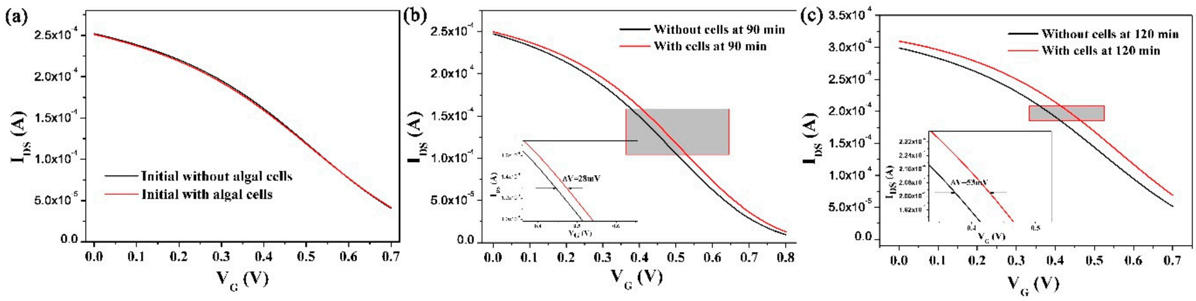

Figure S4. Device characteristics in the culture medium with the salt and light irradiation with and without algae cells at typical time points of (a) 0 min, (b) 90 min and (c) 120 min.

From Figure S4a, there was no difference in the OECT response under the combined treatment with and without the algal cells, as there was no cell attachment on the device initially. At time point of 90 min, as shown in Figure S4b, some cells had precipitated and attached on the device under the combined treatment. The attached cells caused the transfer curve a shift to higher gate voltage, with a 13 mV change compared with the response without algal cells. At time point of 120 min, as shown in Figure S4c, there was a lager shift because of the completion of cells settlement, with a shift value of 53 mV compared with the response without algal cells. These results manifested that the change of the OECT response was caused by the cells' attachment.

The I_{DS} vs V_G curve in buffer with $NaHCO_3$ was measured, and the result was shown in Figure S5. As shown in Figure S5, the transfer characteristic shifted to a larger gate voltage in a three-minute delay too, with a voltage change measured as 52 mV. This shift was also caused by the settlement of green cyst cells on the OECT channel film. Because the comparison was made before and after the algal cells' attachment, in which the OECTs were in the same solution condition with $NaHCO_3$, the influence of $NaHCO_3$ on the device performance was excluded.

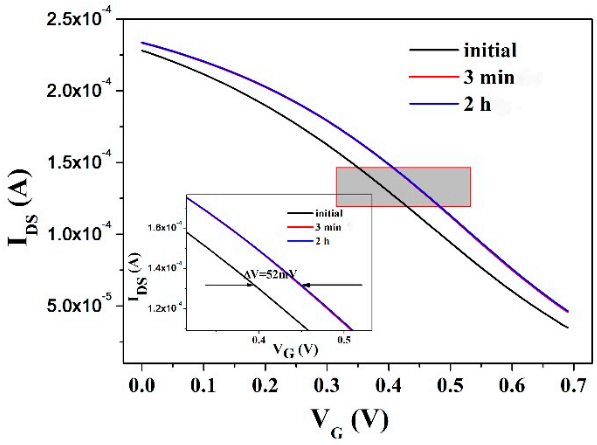

Figure S5. Transfer characteristics of the OECT in the culture medium with sodium bicarbonate initially and at 3 min and 2 h after loading the green cyst *H. pluvialis* cells, V_{DS} = 0.1 V.

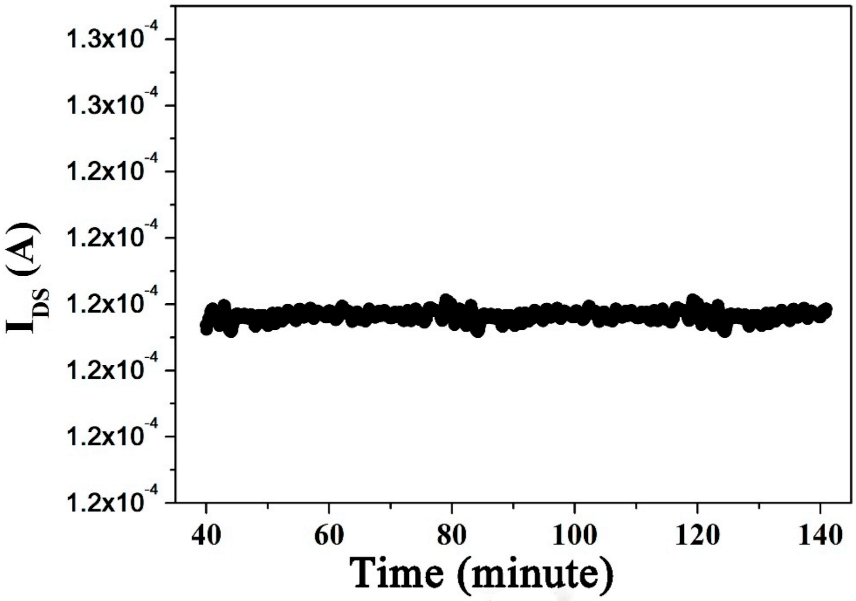

Figure S6. In situ OECT response with *H. pluvialis* cells for 2 h without the light and salt treatment, V_G = 0.3 V, V_{DS} = 0.1 V.
